# *direct* Stochastic Optical Reconstruction Microscopy to Determine the Oligomeric State of Proteins on the Plasma Membrane and Their Accessibility for Immunotherapeutic Antibodies

**DOI:** 10.21769/BioProtoc.5767

**Published:** 2026-08-05

**Authors:** Patrick Eiring, Sören Doose, Nele Bauer, Markus Sauer

**Affiliations:** 1 Department of Biotechnology and Biophysics, Biocentre, University of Würzburg; Würzburg, Germany; 2 Rudolf Virchow Center, Research Center for Integrative and Translational Bioimaging, University of Würzburg; Würzburg, Germany

**Keywords:** Single-molecule localization microscopy, Super-resolution fluorescence microscopy, Membrane proteins, Live-cell staining, Immunofluorescence, Immunotherapy

## Abstract

Super-resolution fluorescence microscopy enables the visualization of protein structures at nanometer resolution, providing insights into receptor organization on the plasma membrane that are essential for the development and optimization of immunotherapies. In this context, monoclonal antibodies are employed, which typically bind only a subset of available membrane receptors, due to steric hindrance or otherwise limited epitope accessibility, to quantify the accessible targets. These accessible targets, rather than the total receptor density, are critical for determining therapeutic efficacy. Here, we present a simplified, robust protocol to quantify antibody-accessible endogenous receptors using monoclonal antibodies directly labeled with fluorescent dyes in combination with total internal reflection fluorescence (TIRF) *direct* stochastic optical reconstruction microscopy (*d*STORM). The method employs optimized labeling and fixation conditions to preserve the native receptor distribution, enabling precise quantification of accessible receptors and their stoichiometry at single-molecule resolution. Omitting secondary antibodies and minimizing fixation-induced artifacts prevents artificial clustering and maintains the physiological binding pattern of therapeutic antibodies. The standardized workflow delivers therapy-relevant information about receptor accessibility and organization underlying therapeutic antibody binding, thereby advancing the mechanistic understanding of immunotherapy resistance and personalized treatment strategies across diverse membrane protein targets.

Key features

• Influence of fixation conditions on receptor epitope accessibility.

• Use of therapeutic antibodies for quantitative estimation of receptor availability relevant to immunotherapy.

• Fluorophore localizations provide information on antibody binding events below the optical resolution limit.

## Graphical overview


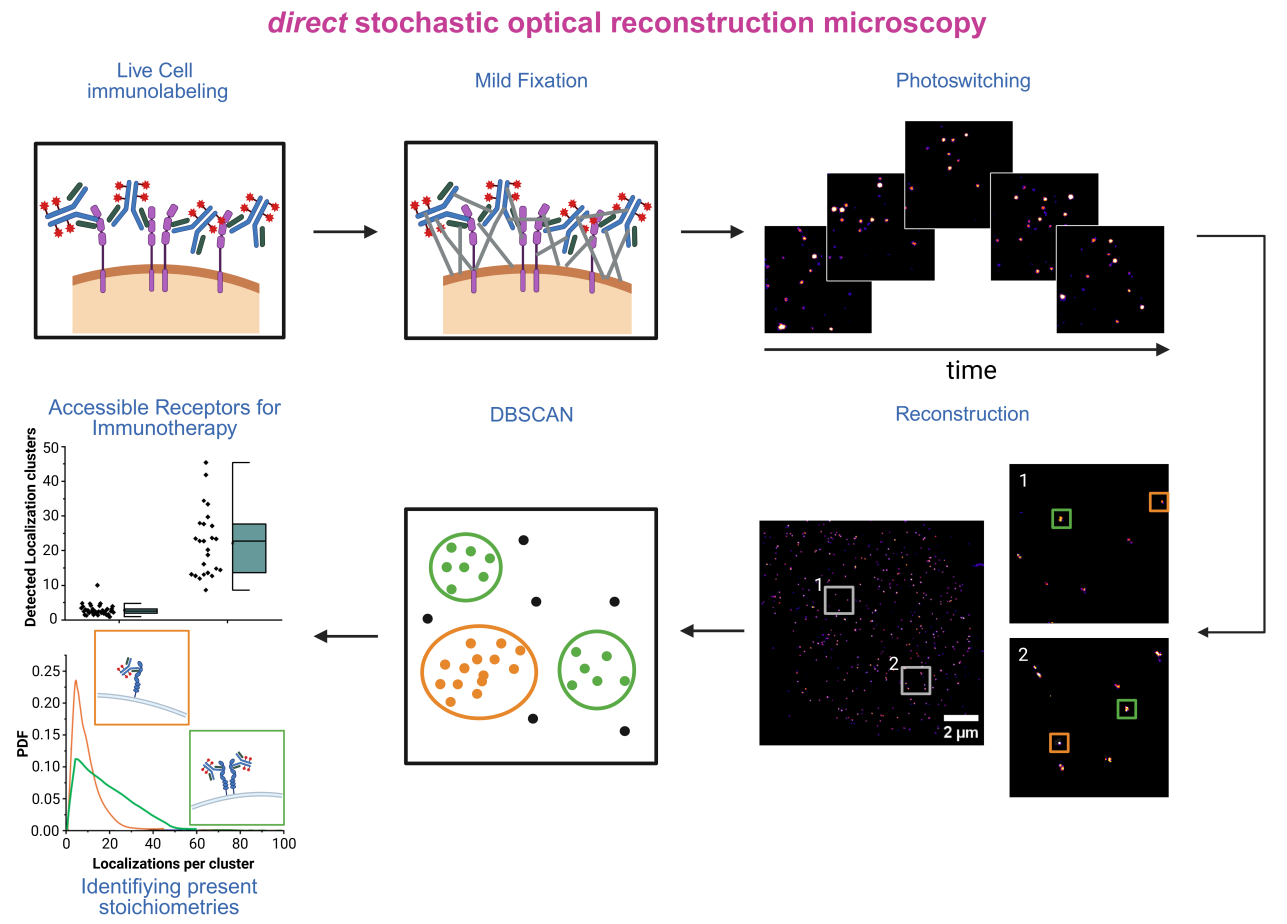


**Graphical overview of the *direct* stochastic optical reconstruction microscopy *(d*STORM)-based workflow for quantification of antibody-accessible endogenous membrane receptors.** The schematic summarizes the complete workflow, including live-cell immunolabeling, fixation, imaging using *d*STORM, and analysis with combined rapi*d*STORM and Python-based tools like LOCAN and DBSCAN.

## Background

Advances in immunofluorescence microscopy, particularly super-resolution techniques, have enabled the visualization of molecular structures far below the classical diffraction limit of ~200 nm [1,2]. Membrane receptors are of special interest in such studies, as they mediate essential cellular processes such as signal transduction, adhesion, and metabolic regulation [3,4]. Because receptor malfunction contributes to a wide range of diseases, membrane proteins have become central targets in biomedical research and therapeutic development [5]. In particular, personalized immunotherapies have established membrane receptors as key targets in the treatment of autoimmune disorders and, even more prominently, for combating various malignancies, including B-cell lymphomas and multiple myeloma [6–10].

For these applications, precise quantification of antibody-addressable receptor densities on the cell membrane and their nanoscale organization is essential, as therapeutic efficacy depends not only on overall receptor quantities but also on accessibility, spatial distribution, and local stoichiometry. However, this information remains largely inaccessible to standard clinical methods. For example, flow cytometry enables rapid analysis of large cell populations but fails to detect low-expressed receptors (<1,000 per cell) and provides no spatial or organizational information [11,12]. In contrast, super-resolution microscopy techniques, especially *direct* stochastic optical reconstruction microscopy (*d*STORM), resolve these limitations by offering nanometer precision and molecular quantification using the same antibodies as employed in therapeutic targeting [13–16]. By correlating receptor density and organization with treatment outcomes across samples, *d*STORM-based imaging can serve as an early analytical indicator of antibody treatment efficacy.

Despite these promises, quantitative *d*STORM of membrane proteins remains technically challenging because standardized workflows for labeling, fixation, and data interpretation are still missing. Having a validated workflow available is particularly important when using monoclonal antibodies that are identical or closely related to widely used clinical therapeutic antibodies, as such antibodies preserve the native epitope specificity of the therapeutic setting. Typical immunofluorescence protocols use secondary antibodies that can dramatically induce artificial clustering via multivalent crosslinking and thus may distort data interpretation [17]. Erroneous secondary antibody effects can be minimized by prefixation, a strategy commonly used in immunofluorescence, which often leads to epitope masking or membrane artifacts [18]. Therefore, live-cell staining with labeled primary antibodies followed by mild fixation offers the most effective compromise between structural preservation and labeling efficacy for quantitative *d*STORM analysis.

Subsequent data analysis combines single-molecule localization with density-based clustering algorithms. Quantitative analysis of localization cluster properties yields information on spatial distribution, heterogeneity, and average receptor molecules per cluster (so-called stoichiometry) of membrane receptors. The analysis scheme enables estimation of receptor cluster stoichiometries [19,20] and therefore helps with detecting immunotherapy-relevant trends despite steric limitations that prevent the determination of absolute stoichiometric ratios.

This protocol provides a detailed workflow for quantifying therapy-relevant receptor densities and nanoscale receptor organization. It outlines critical steps for immunolabeling, *d*STORM imaging, and data quality control and includes guidelines for DBSCAN-based analysis in the context of clinically relevant receptor assessment. The protocol offers a standardized approach for investigating antibody-accessible membrane receptors in mechanistic studies of receptor organization with applications for immunotherapy research and clinical decision support.

## Materials and reagents

**Biological materials**

1. Cell lines or primary cells expressing the membrane proteins to analyze

*Note: The protocol was tested and validated on the following cell lines: Raji, Jurkat, OPM-2, RPMI-8266, MM1.S, HEK293T, and COS-7, as well as primary B and T and multiple myeloma cells.*

**Reagents**

1. Fetal bovine serum (FBS) (Merck, catalog number: F7524); store at -20 °C

2. Penicillin-streptomycin solution (Merck, catalog number: P4333); store at -20 °C

3. RPMI1640 (Merck, catalog number: R8758); store at 4 °C

4. 1× phosphate-buffered saline without calcium and magnesium (PBS) (Merck, catalog number: D8537); store at 4 °C

5. Therapeutic antibody (e.g., anti-CD38 antibody Daratumumab) or antibody of interest, either unconjugated or conjugated to AF647 at a degree of labeling (DOL, or molar dye:antibody ratio) between 2 and 4

6. Alexa Fluor 647 (AF647) NHS-ester (N-hydroxysuccinimide ester) (Thermo Fisher Scientific, catalog number: A20006); store at -20 °C

7. Dimethylsulfoxide (DMSO) (Thermo Fisher Scientific, catalog number: D12345); store at room temperature (RT)

8. Sodium bicarbonate (NaHCO_3_) (Thermo Fisher Scientific, catalog number: 11428856); store at RT

9. Cysteamine hydrochloride (MEA) (Merck, catalog number: M6500); store at 4 °C under inert gas

10. Potassium hydroxide (KOH) (Merck, catalog number: 1.05032.1000); store at RT

11. Formaldehyde (Merck, catalog number: F8775); store at RT

12. Glutaraldehyde (EMS, catalog number: 16220); store at RT

13. Poly-D-lysine (PDL) (optional) (Merck, catalog number: P6407); store at -20 °C

14. Sodium azide (NaN_3_) (Merck, catalog number: S2002-25G); store at RT

**Solutions**

1. Supplemented RPMI1640 (see Recipes)

2. Labeling buffer (see Recipes)

3. Antibody storage buffer (see Recipes)

4. Poly-D-lysine solution (see Recipes)

5. Staining solution (10 μg/mL antibody) (see Recipes)

6. Fixation solution (see Recipes)

7. *d*STORM switching buffer (pH 7.4) (see Recipes)

**Recipes**

**1. Supplemented RPMI1640**

**Table:** 

Reagent	Final concentration	Quantity or volume
RPMI1640	n/a	500 mL
FBS	~10%	50 mL
Penicillin-streptomycin solution	~1%	5 mL
Total	n/a	555 mL

In our example, we use multiple myeloma cell lines. Depending on the cells used, the cell medium may vary.Please note that cell culture should generally be performed in a biosafety cabinet and that all cell culture solutions should be sterile. Depending on the cell type and handling conditions, penicillin-streptomycin may be added.

**2. Labeling buffer**

**Table:** 

Reagent	Final concentration	Quantity or volume
ddH_2_O	n/a	20 mL
NaHCO_3_	200 mM	336.04 mg
Total	200 mM	20 mL

Dissolve 336.04 mg of NaHCO_3_ in 20 mL of ddH_2_O to get a 200 mM solution. Check if the pH is around 8.3–8.7. If not, verify the quality of the NaHCO_3_ and replace it if necessary. The buffer can be stored at 4 °C for several months.

**3. Antibody storage buffer**

**Table:** 

Reagent	Final concentration	Quantity or volume
1× PBS	n/a	50 mL
NaN_3_	0.02%	10 mg
Total	0.02%	50 mL

Dissolve 10 mg of NaN_3_ in 50 mL of 1× PBS to get a 0.02% solution. The buffer can be stored at 4 °C for several months.

**4. Poly-D-lysine solution**

Dissolve 5 mg of PDL in 50 mL of ddH_2_O.

**5. Staining solution**

Dilute the AF647-labeled antibody to a final concentration of 10 µg/mL in 200 µL of supplemented RPMI1640 to prepare the staining solution for one well. If multiple wells are stained, adjust the total volume accordingly.

**6. Fixation solution**

**Table:** 

Reagent	Final concentration	Quantity or volume
1× PBS	n/a	1,864 μL
36%–38.5% formaldehyde solution	~2.3%	120 μL
25% glutaraldehyde solution	0.2%	16 μL
Total	n/a	2,000 μL

Besides using formaldehyde as a fixative, the addition of glutaraldehyde is recommended for better immobilization and crosslinking of the proteins. The fixation solution should be prepared in a fume hood, as concentrated formaldehyde and glutaraldehyde are harmful.

**7. *d*STORM switching buffer (pH 7.4)**

**Table:** 

Reagent	Final concentration	Quantity or volume
1× PBS	n/a	4,900 μL
MEA (5 M)	100 mM	100 μL
KOH (5 M)	n/a	8 μL
Total (optional)	n/a	5,008 μL

First, prepare a 5 M MEA stock solution: Dilute 568.05 mg of MEA in 1 mL of 1× PBS. The MEA stock solution can be stored at -20 °C for at least 3 months. For the *d*STORM switching buffer, add 100 μL of the 5 M MEA to 4,900 μL of 1× PBS to generate a 100 mM solution. Adjust the pH with ~8 μL of 5 M KOH to 7.4–7.5 at room temperature.

*Note: The amount of KOH needed for pH adjustment may vary. It is crucial that the pH of the switching buffer lies within this range. Slight deviations may lead to inefficient removal of oxygen or unreliable photoswitching behavior.*

**Laboratory supplies**

1. T25 cell-culture flasks (Sarstedt, catalog number: 83.3910.502)

2. 10 mL serological pipettes

3. 1.5 mL reaction tubes (Safe-Lock)

4. 15 mL conical tubes

5. 10 μL pipette tips

6. 200 μL pipette tips

7. 1,000 μL pipette tips

8. 8-well or 18-well imaging chamber with high precision coverslip (Cellvis, model: C8-1.5P)

9. 40 kDa Zeba spin desalting columns, 0.5 mL volume (Thermo Fisher Scientific, catalog number: A57760)

10. Cell counter (Logosbio, model: LUNA-FX7, catalog number: L70001)

## Equipment

1. LAQUAtwin pH-11 pH-Meter (Horiba Europe GmbH, catalog number: 895650)

2. Centrifuge (Thermo Scientific, model: Heraeus Fresco 21)

3. Nanophotometer (Implen GmbH, Brand, catalog number: P300)

4. Custom-made *d*STORM microscope based on an Olympus IX-71 inverted microscope body with TIRF illumination and a high NA oil-immersion objective (60×, NA 1.45; Olympus)

5. Water bath (37 °C) (GFL, model: 1003, catalog number: 46685)

6. CO_2_ incubator (Binder, model: KT053, catalog number: 9020-0311)

7. Class II biological safety cabinet (Heraeus, model: HeraSafe HS12, catalog number: 1511)

8. Pipetboy (Brand, model: accu-jet S)

9. Racks for 1.5 mL reaction tubes and for 15 and 50 mL conical tubes

10. Refrigerator

11. Box with ice

## Software and datasets

1. rapi*d*STORM (https://stevewolter.github.io/rapidSTORM, GPL-3.0 license, free access)

2. Python 3.10, 3.11, or 3.12 (https://www.python.org, Python Software Foundation License Version 2, free access)

3. Jupyter Lab (https://github.com/jupyterlab/jupyterlab, BSD-3-Clause license, free access)

4. Locan (https://github.com/super-resolution/Locan, BSD-3-Clause license, free access)

5. napari (https://napari.org, BSD-3-Clause license, free access)

6. napari-locan (https://github.com/super-resolution/napari-locan, BSD-3-Clause license, free access)

7. OriginPro2023b (Origin2023b, https://www.originlab.com) (paid license required)

Example data and code have been deposited to GitHub: https://github.com/super-resolution/Eiring-et-al-2025-supplement (BSD-3-Clause license).

## Procedure

**A. Fluorescence labeling of the antibody with Alexa Fluor 647**

This section describes the fluorescence labeling of antibodies by coupling primary amines with NHS-ester reactive organic fluorophores. In this example, 50 μg of an IgG antibody is coupled with 6 μg of AF647-NHS, resulting in a DOL of ~3. For quantitative analysis, we recommend using AF647, as it shows the most reliable photoswitching and target detection compared with other fluorophores.

*Note: Section A may be skipped if a commercial antibody already coupled with AF647 (degree of labeling between 2 and 4!) has been purchased.*

**Critical:** Before coupling the target protein or antibody with NHS-ester reactive dyes, check that the protein solution is free of BSA or glycerol, as these molecules are not efficiently removed by the columns. Target proteins in PBS or PBS supplemented with NaN_3_ are best suited.

**Figure 1.**
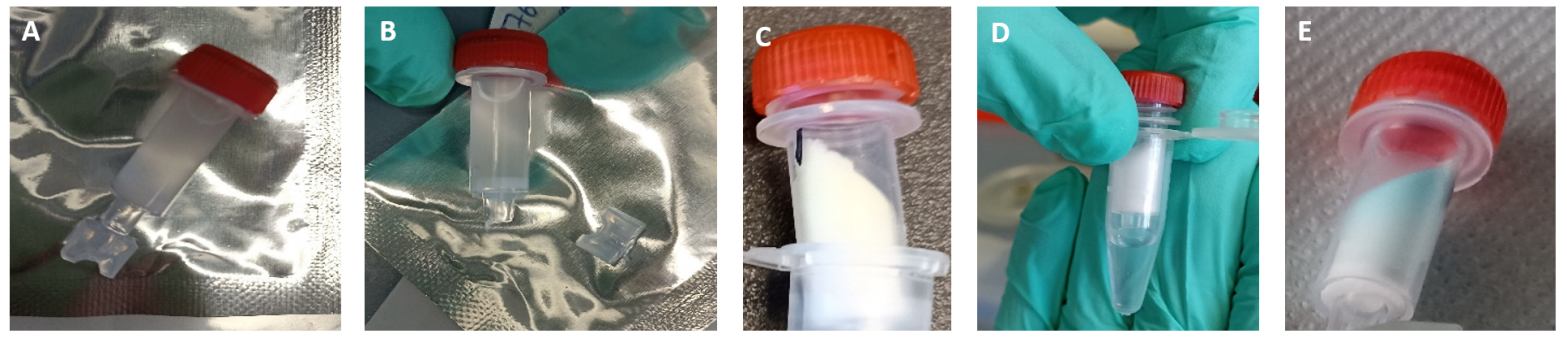
Buffer exchange and removal of excess NHS esters using Zeba Spin desalting columns. (A, B) Images showing the column before (A) and after (B) the removal of the bottom cap. (C) Image showing the gradient of the matrix bed after centrifugation. (D) After centrifugation, ensure that the solution ran completely through the column and that enough space is left in the tube. (E) Unbound dye will be trapped in the column. Make sure the dye does not fully run through the column.

1. Bring all necessary reagents and tubes to room temperature.

2. Prepare the Zeba Spin desalting columns by marking their position (as in **Figure 1C**). The columns must always be placed in the centrifuge in the same orientation so that a slanted column bed is formed.

3. Unscrew the bottom cap of the column and transfer the Zeba Spin desalting column into a 1.5 mL reaction tube. Do not close the lid completely and centrifuge at 1500× *g* for 1 min.

**Critical:** The screw cap should be completely removed and only loosely screwed on to allow the buffer to flow through (**Figure 1A, B**).

4. Discard the filtrate, place the desalting column back into the reaction tube, and slowly pipette 300 μL of 100 mM NaHCO_3_ onto the column bed.

**Critical:** The column bed should remain slanted and intact (**Figure 1C**).

5. Centrifuge at 1,500× *g* for 1 min and discard the filtrate (**Figure 1D**).

6. Repeat steps A4–5 a total of three times.

7. Dab the bottom of the desalting column, transfer it to a new 1.5 mL reaction tube, and slowly pipette 70–200 μL of antibody solution (containing 50 μg total protein) onto the column bed. Centrifuge at 1,500× *g* for 2 min. Keep the filtrate, as it contains the antibody.

**Critical:** Keep the filtrate and, if necessary, re-determine protein concentration in NaHCO_3 _buffer.

8. Meanwhile, prepare AF647-NHS ester: Centrifuge the dye tubes briefly to collect any residue at the bottom of the tube.

Dissolve the AF647-NHS ester in fresh DMSO (anhydrous) to a final concentration of 10 g/L. Resuspend at least 20 times.

*Note: Since only a small amount of dye is needed, aliquoting followed by vacuum drying is recommended to avoid hydrolysis of the NHS group.*

9. Add 0.6 μL of AF647-NHS ester (6 μg) to the antibody solution (50 μg) and mix thoroughly. Then, shake gently for 30–60 min protected from light (e.g., at 60 rpm).

*Note: Depending on the dye and protein used, varying amounts of NHS-ester reactive dyes may be needed. The labeled antibody should have a degree of labeling between 2 and 4.*

10. Approximately 15 min before the end of the reaction, prepare new desalting columns for the storage buffer.

11. As in step A2, mark the position of the Zeba Spin desalting column. The column must always be placed in the centrifuge in the same orientation.

12. Unscrew the bottom cap and transfer the Zeba Spin desalting column to a 1.5 mL tube. Do not close the lid completely and centrifuge at 1,500× *g* for 1 min.

**Critical:** The screw cap should be completely removed and only loosely screwed on to allow the buffer to flow through.

13. Discard the filtrate, place the column back into the reaction tube, and slowly pipette 300 μL of 1× PBS or 1× PBS + 0.02% NaN_3_ onto the column bed.

**Critical:** The column bed should remain slanted and intact. Centrifuge at 1,500× *g* for 1 min and discard the filtrate.

14. Repeat step A13 a total of three times.

15. Dab the bottom of the desalting columns, transfer it to a new 1.5 mL reaction tube, and slowly pipette the antibody– AF647-NHS solution onto the column bed.

16. Centrifuge at 1,500× g for 2 min and keep the filtrate.

**Critical:** The dye should not have passed completely through the column (**Figure 1E**); otherwise, free AF647-NHS ester may still be present. If this occurs, prepare a new spin column (repeat steps A11–14) and purify the antibody–AF647-NHS solution again.

17. To determine the final antibody concentration and degree of labeling, use the same storage buffer for calibration of the nanodrop. Mix the antibody solution gently before measuring.

18. The formula to determine the degree of labeling from absorbance measurements is as follows:

$$protein\;concentration\;\left(M\right)=\frac{A_{280}-(A_{max}\times CF)}{\varepsilon }\times dilution\;factor$$

$$degree\;of\;labeling\;\left(DOL\right)=\frac{A_{max}}{\varepsilon^{´}\times protein\;concentration\;(M)}\times dilution\;factor$$

A_280_ is the absorbance at 280 nm;

A_max_ is the absorbance of the dye solution measured at the wavelength of maximum absorbance;

CF is the correction factor; adjust for the amount of absorbance at 280 nm contributed by the dye (CF = 0.03 for AF647);

ε is the molar extinction coefficient of the protein at 280 nm;

ε´ is the molar extinction coefficient of the fluorescent dye at the wavelength of maximum absorbance;

The dilution factor is the extent to which the protein:dye sample was diluted for the absorbance measurements.

**B. Staining and fixation of cells using a therapeutic antibody**

This section describes the preparation of antibody-treated cells for microscopic investigation.

*Note: This protocol describes the staining of non-adherent cell lines that are relevant for hematological malignancies (e.g., Jurkat, Raji, or OPM-2 cells). For adherent cell lines, it is recommended to seed cells directly onto coverslips and allow them to spread. The staining can also be performed at 37 °C; however, this could also induce endocytosis and other downstream effects caused by the antibody binding. A comparison with staining at 4 °C may provide further information about internalization kinetics and antibody-induced changes.*

1. Maintain cells in culture with the concentration recommended by cell culture databases in a biosafety cabinet (e.g., DSMZ or ATCC) and split them accordingly.

2. Prepare an 8-well imaging chamber (or equivalent) with PDL. Add 150 μL of PDL solution (0.1 mg/mL) for 1 h at room temperature. Remove the solution, rinse once with ddH_2_O, and allow the wells to air-dry under a biosafety cabinet to avoid contamination.

*Note: The prepared imaging chambers can be stored at 4 °C for 1–2 weeks. PDL coating is not needed for adherent cells.*

3. Seed 2.5 × 10^5^ cells per well and let them adhere to the PDL-coated surface for 1 h in a 37 °C incubator. Check adherence of the cells by gently tipping the chamber to ensure cells have settled and are attached to the surface.

4. In the meantime, prepare the staining solution in 1.5 mL Eppendorf tubes. Add 200 μL of ice-cold 1× PBS or cell culture medium and the antibody to the desired concentration (e.g., 10 μg/mL for Daratumumab).

*Note: For a new antibody, titration is strongly recommended to ensure saturation of all accessible epitopes. Commonly tested concentrations are 0.5, 1, 2, 5, and 10 μg/mL.*

5. Keep the 1.5 mL Eppendorf tubes and the 1× PBS on ice.

6. Take the cells out of the incubator and let them equilibrate for 5 min at room temperature. Then, place the 8-well chambers on ice and wait for 5 min.

7. Remove the cell medium and wash the cells once with ice-cold 1× PBS. Add 200 μL of staining solution to the respective wells for 30 min.

*Note: Staining times may vary depending on the cell line and antibody. For most stainings, an antibody incubation time between 30 and 60 min is sufficient.*

**Critical:** Suspension cells are easily washed away, even when they are adherent to PDL-coated surfaces. Be as gentle as possible when washing, removing, or adding solutions. Always place the pipette tip into one corner and add or remove solutions slowly. This also helps to keep the cells distributed more evenly across the surface and prevents them from clustering in one corner.

8. In the meantime, prepare the fixative. Add 1,864 μL of 1× PBS, 120 μL of formaldehyde stock solution, and 16 μL of glutaraldehyde stock solution into a 1.5 mL Eppendorf tube (adjust volumes if you scale your total volume).

9. After 30 min, remove the antibody solution, wash once with ice-cold 1× PBS, and add 200 μL of fixative per well. Remove the sample from ice and allow it to warm to room temperature.

10. After 15 min, remove the fixative and wash three times with 1× PBS.

11. Protect the sample from light and store it at 4 °C in 1× PBS for up to one week or proceed directly with the imaging part.

**C. Quantitative imaging of the cell sample by *d*STORM**

This section describes the imaging of cells by *d*STORM. For further data analysis, the acquired movies must be exported as *.tif files.

1. Prepare the switching buffer directly before starting measurements, as MEA is oxidized by residual oxygen over time and loses its activity as a redox reagent.

*Note: The switching buffer can be used for several hours but should not be reused on the next day [21].*

2. Leave the sample at room temperature to acclimate, remove 1× PBS, and add 500 μL of switching buffer to each well.

3. Put the sample on the stage and focus on the basal membrane of the cells using TIRF illumination and low laser power to avoid photobleaching or photoswitching.

4. Check for drift of the sample.

**Critical:** The setup must be stable in all three dimensions. An additional drift correction step afterward is not recommended and may introduce analysis errors.

5. Start measurements after following this procedure:

a. Increase the laser power to 2.0–2.5 kW/cm^2^ to achieve efficient photoswitching.

b. Briefly turn the TIRF screw through HiLO and Epi-illumination modes to transiently switch molecules in adjacent planes and bleach background fluorescence.

c. Return to TIRF, check that spots are well focused, and start measurements immediately.

**Critical:** Steps C5a–c are time-sensitive. The entire process from increasing laser power to starting the measurement should not take longer than 10 s and should have the least possible variation in overall duration for comparative experiments.

6. Acquire 15,000 frames with an exposure time of 20 ms (~5 min).

**Figure 2.**
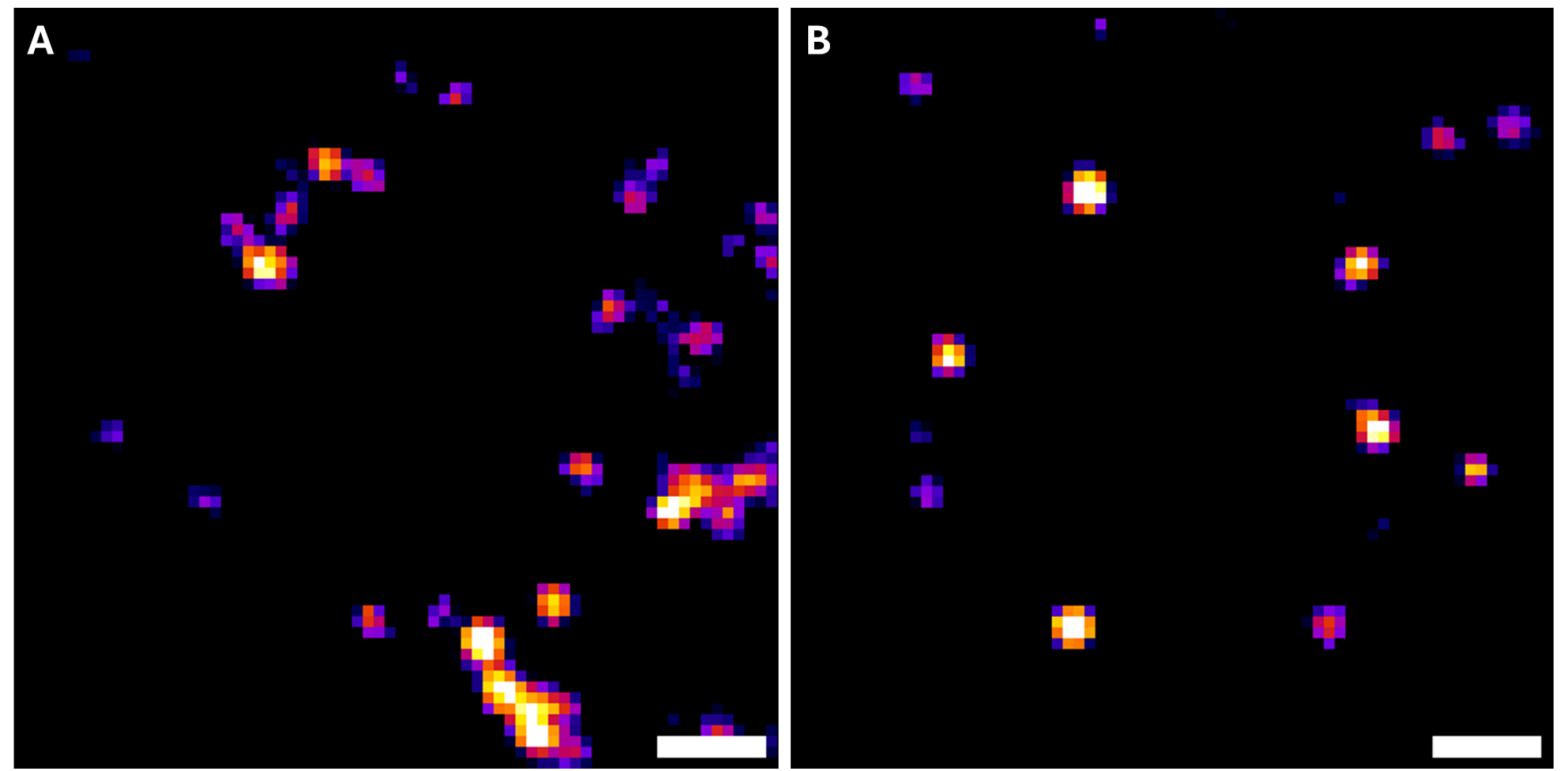
Representative frames of a *direct* stochastic optical reconstruction microscopy *(d*STORM) movie showing overlapping Fluorophore point spread functions (A) and well-separated localizations (B). While the acquired images of panel B will allow quantitative analysis, the *d*STORM movie shown in panel A will result in low localization precision, artifacts, and improper fluorophore separation.

**Critical:** Ensure the setup does not drift during acquisition. Also, visually check during measurements whether fluorophores are well separated to avoid overlapping of neighboring fluorophore PSFs (see **Figure 2**).

7. After image acquisition, repeat steps C4–6 until a sufficient number of cells have been imaged to obtain adequate statistics. (Imaging at least 10 basal cell membranes is recommended; however, if primary cells are used, imaging more cell membranes may be helpful due to the higher variance in receptor expression.)

*Note: For better comparison, always prepare the samples with different conditions in the same multi-well chamber on the same day to minimize differences caused by cell cycle, coating, or buffer conditions.*

## Data analysis

The data analysis pipeline consists of computing single-molecule localizations with the standalone software rapi*d*STORM [22] (this could also be done with alternative software for single-molecule localization) and Python-based localization analysis for localization filtering with regard to photon counts and regions of interest, localization clustering, cluster selection, and analysis of cluster properties. For visualization and exploratory data analysis, we employ the image viewer napari [23] with the napari-locan plugin [24], and Jupyter computational notebooks [25]. For Python-based localization analysis, we employ the Python library locan [20], providing a toolset for localization analysis based on the scientific Python stack (matplotlib, numpy, pandas, scikit-image, scikit-learn, scipy) and the third-party libraries boost-histogram, fast-histogram, lmfit, network, plotly, ray, and shapely.

**A. Processing of the *d*STORM raw data**

This section describes the identification of localizations of single-molecule recordings after image acquisition by *d*STORM.

1. Process the recorded image data (*.tif files) with rapi*d*STORM (or another localization software) to get single-molecule localization data (*.txt files) and reconstruct *d*STORM images (*.png files). Depending on the signal-to-noise ratio in the camera recordings, an intensity threshold must be applied to limit localization data to true fluorophore signals. In our hands, a minimum intensity filter set to 800 photons ensures minimizing false positives and maximizing true positive localizations. *Note: Intensity filtering is applied in rapidSTORM or during further processing of localization data by entering the analog-to-digital counts (ADC) value, which is camera-specific and must be calculated.*

2. Validate that the *d*STORM data is free of drift and has been recorded from the basal membrane throughout the measurements by visual inspection.

3. The *.txt file containing a localization table with localization properties is then visualized by image reconstruction in rapi*d*STORM. Localizations are binned on a grid with pixel sizes of 10 nm in x- and y-dimensions according to their spatial coordinates. Localization counts are rescaled by histogram equalization for appropriate visualization.

4. Alternatively, visual inspection can be done in napari using the napari-locan plugin.

5. The *.txt file containing a localization table is then further analyzed with Python-based scripts or in Jupyter notebooks using the Python library locan together with standard scientific Python libraries.

**B. Set up a Python environment for further analysis steps**

This section describes how to install Python and Python packages in a virtual environment that can be used for further analysis steps. We recommend using uv or conda as the package manager.

1. Use uv (https://docs.astral.sh/uv/) and packages from pypi (https://pypi.org/) to set up a Python virtual environment:

a. Install uv using a standalone installer (https://docs.astral.sh/uv/getting-started/installation/).

b. Update the uv version regularly running “*uv self update*” in a terminal.

c. Open a terminal and enter the project directory by running “*cd <project_directory>*”.

d. Create a virtual environment with Python 3.12 by running “*uv init -p 3.12*”.

e. Add locan by running “*uv add locan[all] pyside6 jupyterlab*”.

f. Add any other dependencies you might want to use by running “*uv add ipywidgets plotly papermill seaborn*”.

g. Alternatively, use the locked versions by copying the pyproject.toml and uv.lock file from https://github.com/super-resolution/Eiring-et-al-2025-supplement into the project_directory and run “*uv sync*”.

2. Alternatively, use packages from conda-forge (https://conda-forge.org/) to set up a Python virtual environment:

a. Use miniforge to download all packages from conda-forge. Download and install miniforge from https://conda-forge.org/download/.

b. Use the miniforge prompt (conda terminal) for all further commands.

c. Create a virtual environment named locan with Python 3.12 by following the instructions provided in the LOCAN documentation at https://locan.readthedocs.io/en/latest/source/installation.html.

d. Activate the locan environment by entering “*conda activate locan*”.

e. Add any other dependencies you might want to use by running “*conda install pyside6 napari napari-locan ipywidgets plotly papermill seaborn*”.

f. For further analysis, use a conda terminal, enter the project directory by running “*cd <project_directory>*”, and activate the locan environment by entering “*conda activate locan*”.

**C. Choosing regions of interest representing individual cell signals**

This section describes the identification of image regions representing individual cells as regions of interest (ROI).

1. Open a terminal and enter the project directory by running “*cd <project_directory>*”.

2. For using uv, there should be a virtual environment installed in the project_directory. For using conda, activate the conda environment and leave out “*uv run*” in the coming commands.

3. Start the ROI selection by running “*uv run locan rois -t 2”*.

Note: When localization data is saved as a *.csv file, e.g., using ThunderSTORM or other localization software, use the command “uv run locan rois -t 4”.

**Figure 3.**
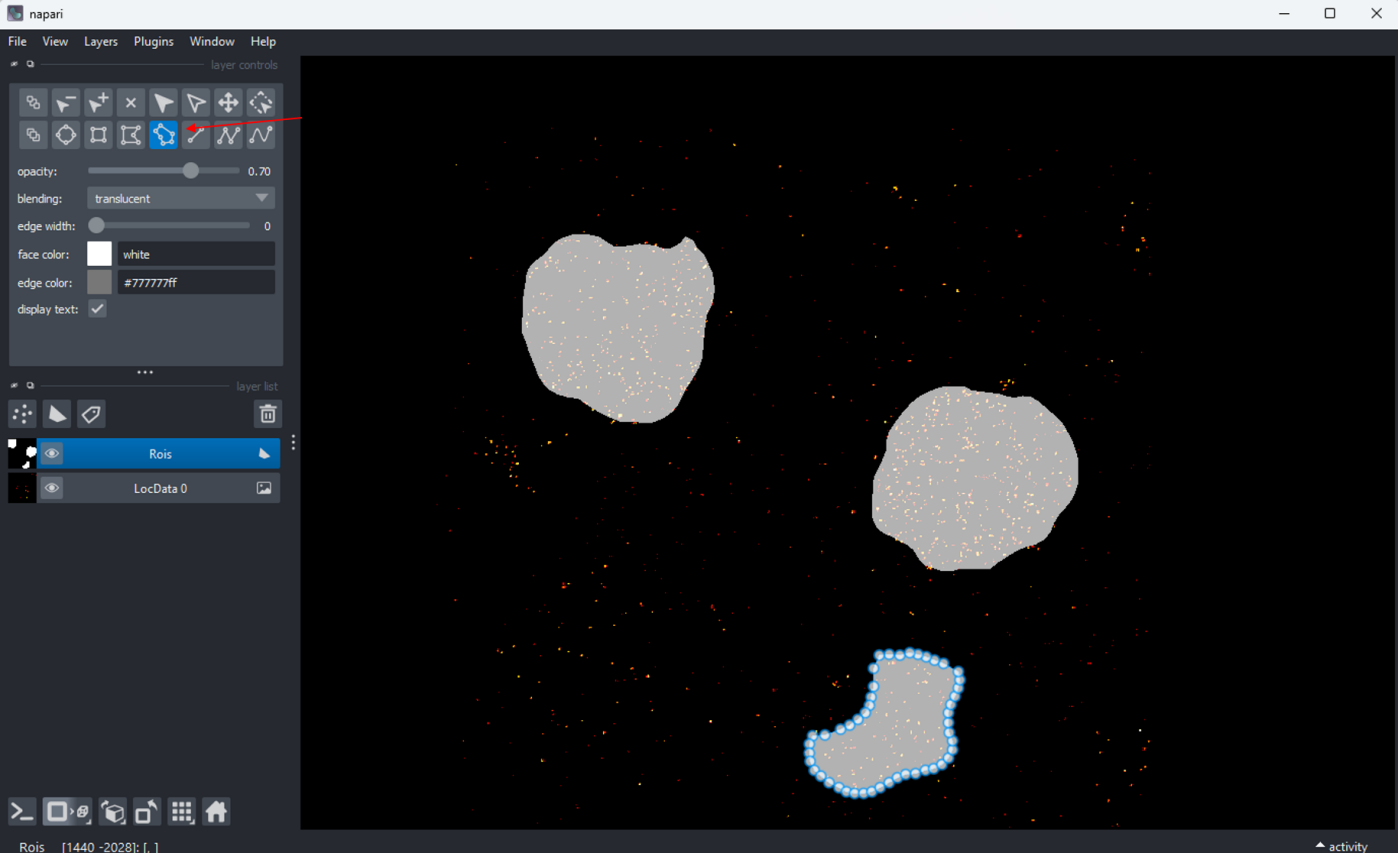
Selection of the region of interest (ROI) for analysis. Multiple basal membrane areas are selected using the *polygon lasso tool* (red arrow) and saved by closing the napari window.

4. Select one *.txt file containing the localization data of one sample.

5. Draw a ROI around the basal membrane area of the cell using the *polygon lasso* or *freehand selection tool*. Avoid overlapping membrane areas or apoptotic cells. If several cells are present in one file, multiple ROIs can be selected (see **Figure 3**).

6. Confirm and save the ROIs by closing the napari window. All ROIs are saved as *.yaml files. Repeat steps C3–6 for all *.txt files.

7. Alternatively, use napari and plugins.

a. Install napari and the napari-locan plugin (might already be installed in the previously used virtual environments).

b. Start napari by running “*uv run napari*” from a terminal.

c. Use napari and the napari-locan plugin to load localization data from the localization file (*.txt file).

d. Create a new shape layer and draw region shapes using the polygon tool.

e. Create and save the region of interest as a *.yaml file using the napari-locan plugin.

**D. Localization analysis for each region of interest**

This section describes Python-based analysis of all localizations within the ROIs representing individual cells.

**Figure 4.**
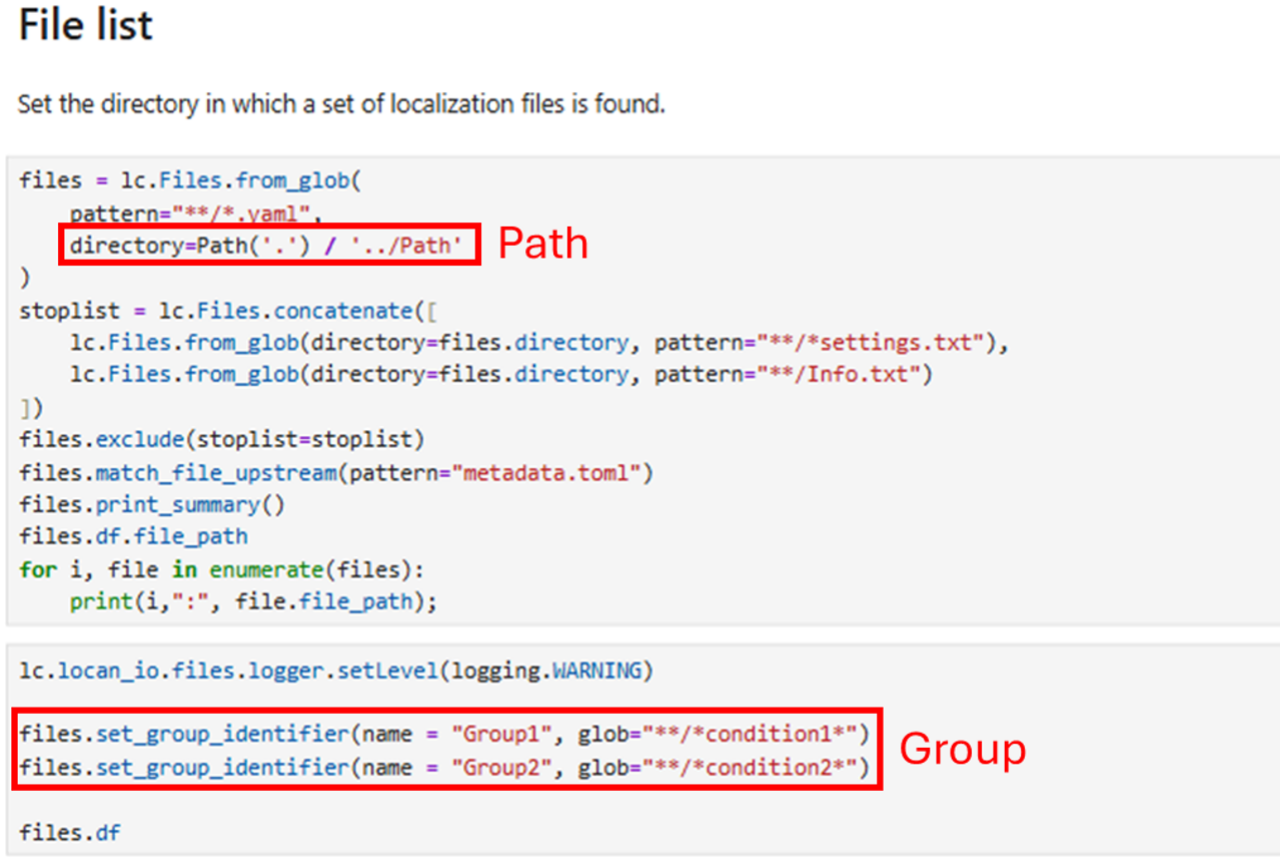
Entering the data path and grouping the data. The folder containing the analyzed *.txt files and their corresponding *.yaml region of interest (ROI) files must be specified under *Path* or *Group*. If multiple conditions are included, the data must be grouped to allow simultaneous analysis.

1. Open a terminal and enter the project directory by running “*cd <project_directory>*”.

2. Copy the example notebooks of the Eiring et al. supplement (https://github.com/super-resolution/Eiring-et-al-2025-supplement/tree/main/notebooks) into the directory “*project_directory/notebooks*”.

3. Start Jupyter lab by running “uv run *jupyter lab*”.

4. Modify and run the notebooks.

5. Select the script “*cluster_analysis_batch*” and enter the path where the *.txt files and their respective *.yaml files can be found (see **Figure 4, Path**).

6. Adjust the following settings: If multiple conditions are analyzed simultaneously, the data belonging together should be grouped, e.g., “*Group1*” or “*Group2*” (see **Figure 4, Group**).

*Note: Files belonging to the same group should contain the same name in their filename or subfolder to allow grouping, e.g., “condition1” or “condition2”.*

**Figure 5.**
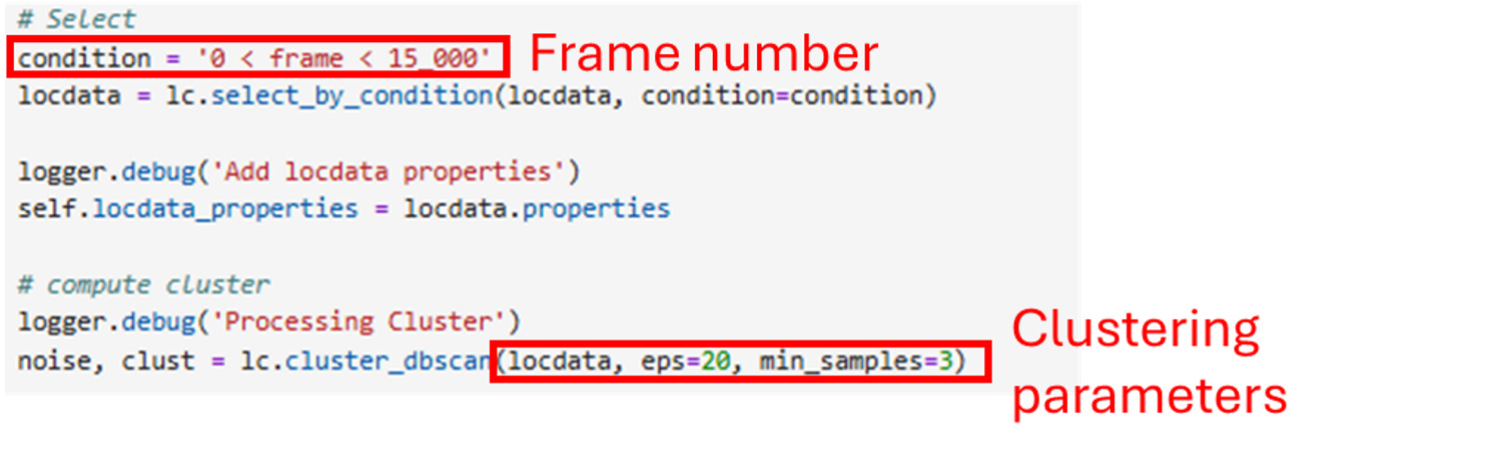
Important parameters for analysis. The frame range was set between 0 and 15,000 frames to ensure that the same time interval was analyzed. For membrane receptor analysis, the DBSCAN clustering parameters were set to an epsilon value of 20 and a min_samples value of 3 localizations per cluster. These parameters provide the most robust analysis of membrane receptors imaged with AF647.

7. Add selection rules for localization if needed. To ensure that all datasets contained the same number of frames, the frame range was set from 0 to 15,000. Additional conditions can be defined for intensity filtering.

8. For cluster detection, the DBSCAN parameter “eps” (the maximum distance value for clustering) was set to 20, and “min_samples” (the minimum number of clustered localizations) was set to 3 (see **Figure 5**). This parameter set provides robust quantification of membrane receptors labeled with AF647 up to localization densities of around 80 μm^-2^ [26].

*Note: When using different fluorophores or when localization precision is lower, these parameters may need to be adjusted accordingly.*

9. Add selection rules for cluster filtering if needed. We applied the condition that a cluster must have more than two localizations to compute a convex hull as the region size (named region_measure_ch).

10. Run the script. The result cells contain information about:

a. *Cluster count:* number of detected clusters.

b. *N_locs_in cluster_relative:* fraction of localizations assigned to cluster relative to the total number of localizations.

c. *Cluster_density:* number of clusters per selected ROI area.

d. *Localization_count_mean:* average number of localizations per cluster.

e. *Region_measure_ch_mean:* average size of the area of the analyzed region.

f. *Localization_density_in_cluster_mean:* average localization density within clusters.

g. *Intensity_sum_mean:* average summed fluorescence intensity per cluster.

h. *Intensity_mean_mean:* average mean fluorescence intensity cluster.

i. *Local_background mean:* average local background signal.

11. While most of the output is primarily supportive and used for quality control, the most important parameters are *Cluster_density* and the *Localization_count_mean.* Save the information about cluster density and localizations per cluster. This data is also saved in a *.csv file and can be used to generate bar graphs or line plots in other software such as Excel or OriginPro.

## Validation of protocol

This protocol has been used and validated in the following research article(s):

• Eiring et al. [27]. Single-molecule localization microscopy reveals the molecular organization of endogenous membrane receptors. *Sci Adv*, 2026, 12(6): p. eaea2310, doi:10.1126/sciadv.aea2310. (Figures 1C–F, 2, 3D–E, and 4G–H)

## General notes and troubleshooting

**General notes**

1. **Quantification limitation.** This protocol quantifies antibody-accessible receptors—rather than total receptor numbers—as steric hindrance and antibody binding affinity limit complete epitope coverage. For immunotherapy applications, this is exactly the number that reflects the antibody’s mode of action efficiency. Therefore, by focusing on addressable targets, the method provides reliable, quantitative, and relevant data.

2. **Antibody selection.** Receptor stoichiometry (monomers, dimers, oligomers, etc.) can only be reliably determined using monoclonal antibodies. Polyclonal antibodies may bind multiple epitopes per receptor, leading to artificial multimerization and misinterpretation of clustering (as shown in [13]).

3. **Oligomer detection limit.** Distinguishing monomers from dimers is feasible when receptors predominantly exist in one state. Low-abundance oligomers may be overseen due to steric constraints. However, small, treatment-induced changes may be detected when comparing conditions with identical monoclonal antibodies.

4. **Antibody-induced endocytosis**. The live-cell staining approach does not preclude antibody-induced endocytosis or signaling-triggered receptor internalization. This is mainly relevant when staining is performed at RT or 37 °C. Staining on ice (4 °C) sufficiently slows endocytosis to prevent substantial receptor internalization during the staining procedure. Comparing quantification data at 4 °C and 37 °C allows estimation of the extent of antibody-induced endocytosis.

**Troubleshooting**

**Problem 1:** Degree of labeling is too low.

Possible cause: Amount of NHS-reactive dye was too low or partially reacted.

Solution: Add more NHS-reactive dye to the antibody with a too low degree of labeling. Repeat the labeling protocol in section A.

**Problem 2:** High amount of background signal.

Possible causes: Antibody concentration is too high or antibody affinity is too low.

Solution: Titrate antibody to determine optimal concentration. Excessive amounts of antibody may bind nonspecifically to the cover glass or surface coating.

**Problem 3:** Cells or fluorophores are visibly moving or shaking.

Possible cause: Fixation was not sufficient.

Solution: Increase fixation time.

**Problem 4:** Single-molecule signals are too dense and provide overlapping PSFs.

Possible causes: Laser power too low or switching buffer too old.

Solution: Renew switching buffer and/or increase excitation power to improve blinking rates.

**Problem 5:** Signal on the cell membranes is too low to distinguish the specific signal from the background.

Possible causes: Low receptor expression, insufficient antibody concentration, or low binding affinity of the antibody.

Solution: Increase antibody concentrations to reach saturating conditions or use an alternative antibody. If the receptor is expressed at a low level, overlaying the fluorescent image with a previously saved brightfield image may help define the ROIs for image analysis.

## Supplementary information

Supporting information for this protocol is available at https://github.com/super-resolution/Eiring-et-al-2025-supplement and includes notebooks and exemplary data.
